# Coupling vibration analysis of heat exchanger tube bundles under different stiffness conditions

**DOI:** 10.1038/s41598-024-53855-x

**Published:** 2024-02-17

**Authors:** Yifang Yin, Zunce Wang, Mingyue Ma, Jinglong Zhang, Yan Xu, Lidong Li, Mingming Ge

**Affiliations:** 1 College of Mechanical Science and Engineering, Northeast Petroleum University, Daqing, 163318 Heilongjiang China; 2 Heilongjiang Key Laboratory of Petroleum and Petrochemical Multiphase Treatment and Pollution Prevention, Daqing, 163318 Heilongjiang China; 3 National Observation and Research Station of Coastal Ecological Environments in Macao, Macao Environmental Research Institute, Faculty of Innovation Engineering, Macau University of Science and Technology, Macao SAR, 999078 China

**Keywords:** Heat transfer tube bundles, Computational fluid dynamics, Fluid-induced vibration, Fluid–structure coupling, Fluid dynamics, Computational science, Mechanical engineering

## Abstract

A two-dimensional tube bundles fluid–structure coupling model was developed using the CFD approach, with a rigid body motion equation and the Newmark integral method. The numerical simulations were performed to determine the vibration coupling properties between various tube bundles of stiffness. Take the corner square tube bundles with a pitch ratio of 1.28 as the research object. The influence of adjacent tubes with different stiffness on the vibration of the central target tube was analyzed. The research results show that the vibration characteristic of tube bundles is affected by the flow field dominant frequency and the inherent frequency of tube bundles. The vibration of adjacent tube bundles significantly impacts the amplitude and frequency of the central target tube. The equal stiffness and large stiffness tubes upstream or downstream inhibit the vibration displacement of the target tube to some extent. The low-stiffness tubes upstream or downstream significantly enhanced the amplitude of the target tube. The findings can be used to provide a basis for reasonable design and vibration suppression of shell-and-tube heat exchangers.

## Introduction

Working as the most widely used heat exchange equipment, the Shell-and-tube heat exchanger has preferable characteristics such as good heat exchange performance and large heat transfer area^1–3^. It can work under high temperatures and pressure, with simple structure, low cost, convenient cleaning, and strong applicability. Heat exchangers occupy a large proportion of industrial investment, accounting for about 30% of the total investment in chemical equipment, 40% of the total investment in oil refining production equipment, and 20% of the total investment in nuclear power plant equipment. The shell-and-tube heat exchangers have the highest application rate in the industrial heat transfer field, accounting for about 70% of the current heat exchange equipment^4,5^.

**Figure 1:**
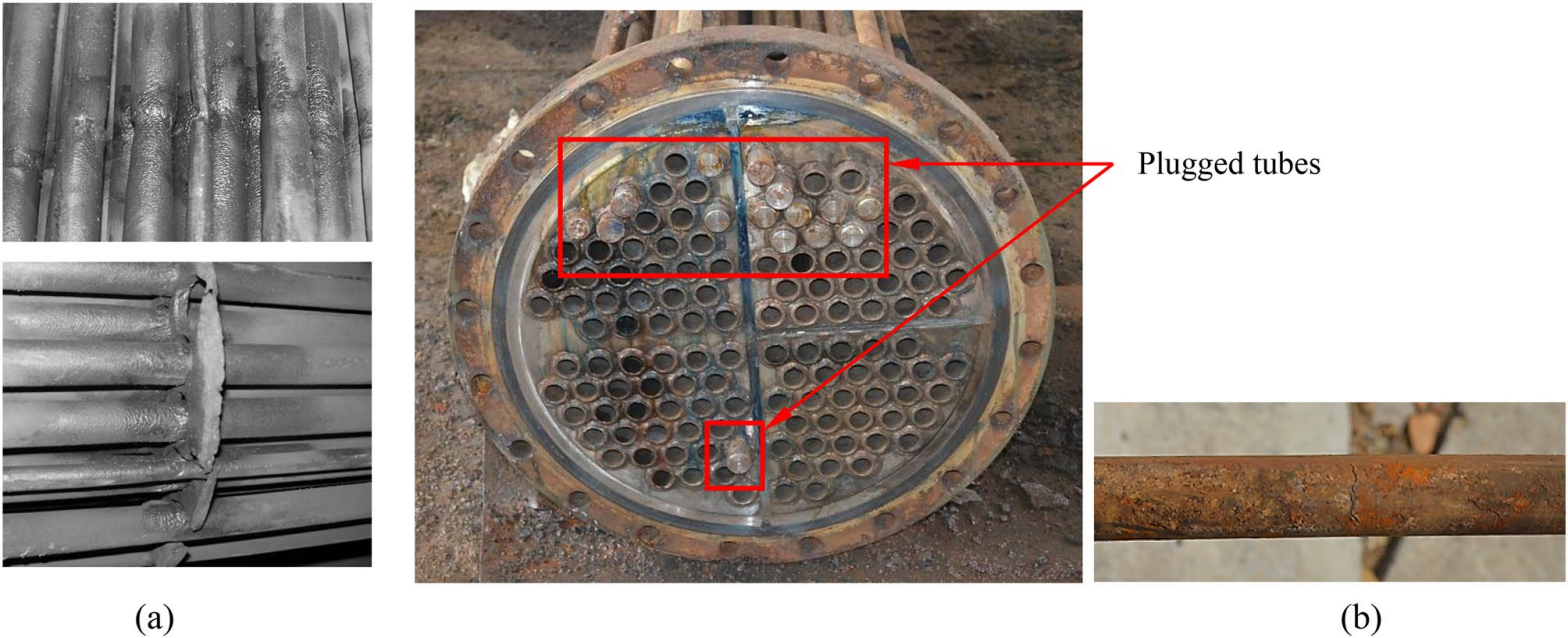
Profiles of failed tube bundles are shown in red rectangular (provided by Daqing Petrochemical Company). (**a**) Touching abrasion between tubes and baffle plate, (**b**) tube fatigue cracking.

For a shell-and-tube heat exchanger, the shell-side fluid scours the tube bundle laterally, leading to a flow-induced vibration, failing the heat exchange tube bundles^6,7^. Researchers have carried out much work in the past decades using theoretical, experimental, and numerical simulation methods. Their work has solved the problem of flow-induced vibration of heat exchanger tubes to a certain extent^8–11^. However, the failure problem caused by coupling vibration between bundles still occurs. Of the 128 heat exchanger tubes in a heat exchanger of Daqing Petrochemical Company, 15 failed and plugged (see Fig. 1). According to the detection and analysis of the failed tubes, the causes of tube failure are the touching abrasion and fatigue generated by the vibration of the tube bundle. The fluid-induced vibration in the heat exchanger is the main factor inducing the vibration, which causes severe economic and engineering losses. Therefore, it is necessary to investigate the coupling vibration between bundles to reduce the harm caused by such problems.

To study the coupling vibration between tube bundles, Tang et al. used numerical simulation to learn the five primary vortex shedding modes of heat exchange tube bundles in two-dimensional unsteady flow under the action of saturated steam fluid, as well as the influence of shedding vortex on lift performance^12–14^, and coupled CFD method with computational structural dynamics (CSD) method. The fluid–structure coupling response can be predicted in the time domain. Tan^15^ et al. established a fluid–structure coupling vibration model to study the critical flow velocity of square-arranged bundles, and the calculated results agree well with the experimental data. Tang et al.^13^ proposed a high-precision, low-cost CFD/CSD high-order coupling algorithm. Fluent software was combined with the dynamic mesh method to systematically study the dynamic response of the structure of the tube bundles in the dense heat exchange tube bundles. Tube drag, lift, frequency, and amplitude were analyzed in the time and frequency domain.

**Figure 2:**
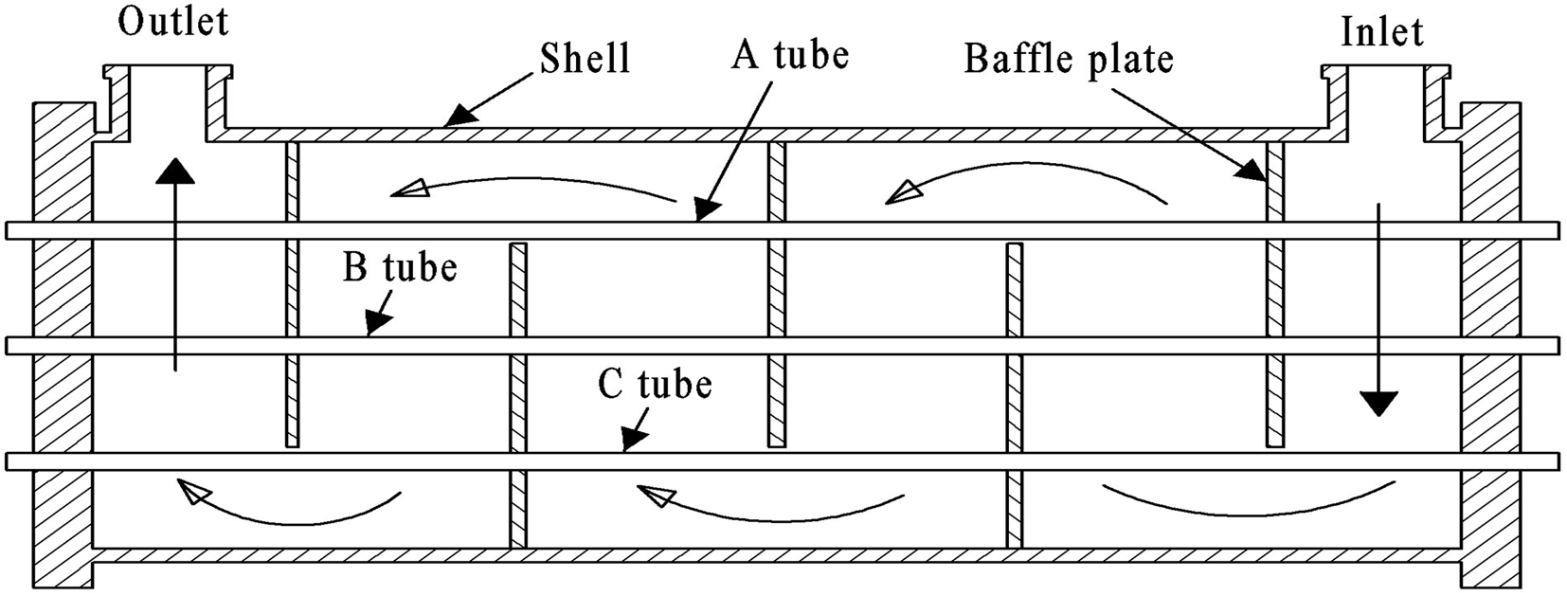
Classification of heat exchange tubes.

In a typical shell-and-tube heat exchanger, the stiffness of tubes is different under different constraints. The tubes can be generally divided into A, B, and C types according to the different number of baffle plates. As shown in Fig. 2, the failure shell and tube heat exchanger have five baffle plates. A heat exchange tube passes through three baffle plates and two baffle gap areas while the stiffness is centered. B type of heat exchange tube through all five baffle plates, the large stiffness. The C type of heat exchange tubes passes through two baffle plates and three baffle notched areas with small stiffness.

The influences of coupling vibration of tube bundles on the central target tube are investigated when the stiffnesses of adjacent tubes are different, especially for the multiple flexibly-mounted tube bundles^16,17^. As shown in Fig. 1, the location of failure heat exchange tubes is the interface area of the two types of tubes, illustrating that the stiffnesses of tube bundles in the transverse flow are quite different. It should be considered when the coupling vibration characteristics between tubes are analyzed.

Fluent software is adopted to calculate the flow between tube bundles numerically. According to the actual cases of tube bundle damage, the vibration behavior of adjacent multiple bundles and the interaction mechanism between tubes were studied considering the non-uniform bundle stiffness combination. The coupling vibration of the central target tube and adjacent tubes with different stiffness was analyzed from the aspects of the flow field, structural response, and the movement path of the tube bundles to reveal the coupling vibration mechanism between non-uniform bundle stiffness.

## Numerical model for vibration of tube bundle under transverse flow

### Calculation method of flow field between tube bundles

Reynolds time-averaged method is used to calculate the transverse flow between bundles. The time-averaged continuity equation and momentum equation in the form of the tensor are as follows^18^:

1 $$\frac{\partial \rho }{{\partial t}} + \frac{\partial }{{\partial x_{j} }}\left( {\rho u_{j} } \right) = {0}$$

2 $$\frac{{\partial u_{i} }}{\partial t} + \frac{\partial }{{\partial x_{j} }}(\rho u_{i} u_{j} ) = - \frac{\partial p}{{\partial x_{i} }} + \frac{\partial }{{\partial x_{j} }}(\mu \frac{{\partial u_{i} }}{{\partial x_{j} }} - \rho \overline{{u^{\prime}_{i} u^{\prime}_{j} }} )$$

It changes velocity and other variables into a time average, where the extra term is Reynolds stress, representing turbulence's influence. The turbulence model needs to be introduced to make the time-averaged equation closed.

### Turbulence model

Due to the small gaps between tubes, the fluid velocity in the main flow area significantly differs from the velocity between the gaps. Meanwhile, the flow around produces a large number of vortex structures. Therefore, it is essential to select a suitable turbulence model to simulate the flowfield between dense tube bundles to describe the interaction between the fluid and structure honestly.

Researchers have used different turbulence models to numerically calculate the flow field induced by tube beam vibration. For example, Bao^19^ used RNG k–ε to calculate the three-dimensional flow field between tube bundles with a pitch ratio 1.28. By comparing with the experimental data, Zhao^20^ believed that the Transition SST model is more suitable for numerical simulation of turbulent flow field between close-packed tube arrays. Darwish^21^ stated that the SST k–ω was more reliable in solving the flow between a rotated square tube array.

**Figure 3:**
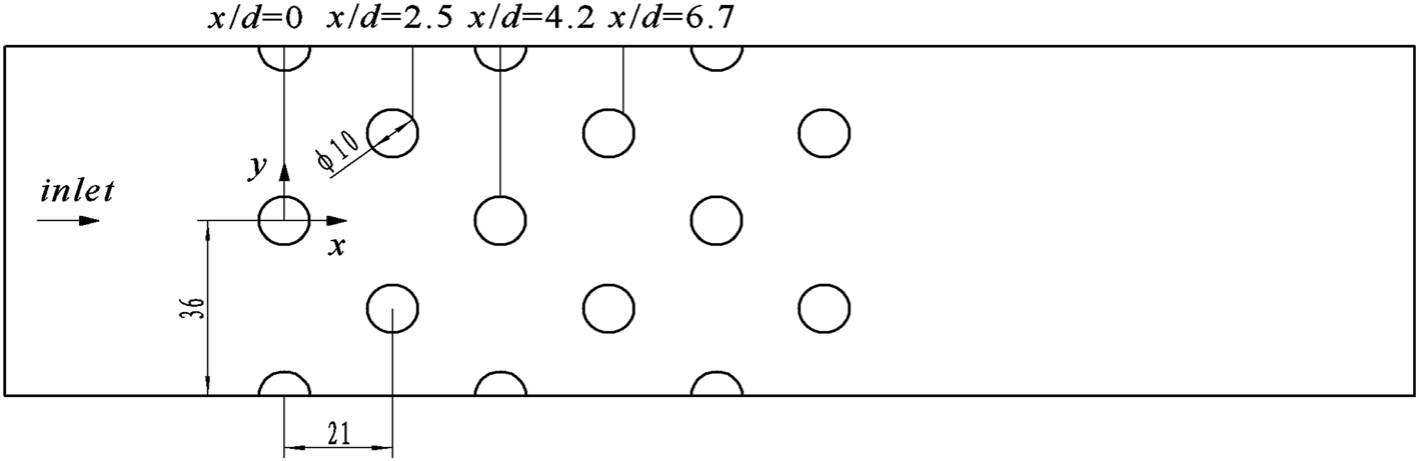
The geometric model for turbulence model verification.

**Figure 4:**
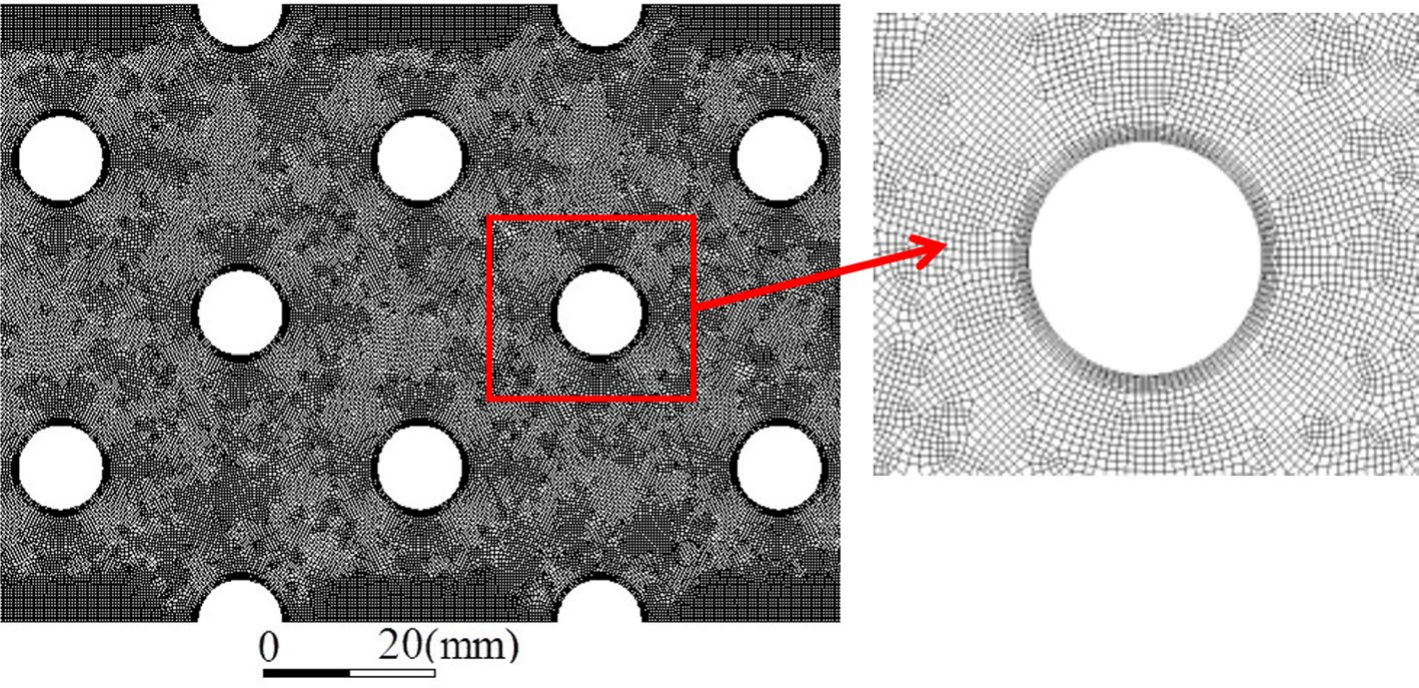
Zoom on the numerical mesh with scales.

The turbulence model selected will be verified by comparing the computational results of different turbulence models with experimental data. Based on the velocity field experimental data of the staggered bundles of Balabani^22^, as shown in Fig. 3, a two-dimensional calculation model was established. The calculation mesh is shown in Fig. 4. There are 108 nodes on the tube bundle boundary. The number of encryption layers was 6, the initial height was 0.2 mm, and the mesh growth rate was 1.1. The RNG k–ε, SST k–ω, and Transition SST model were adapted for numerical simulation, and the velocity field distribution was obtained.

**Figure 5:**
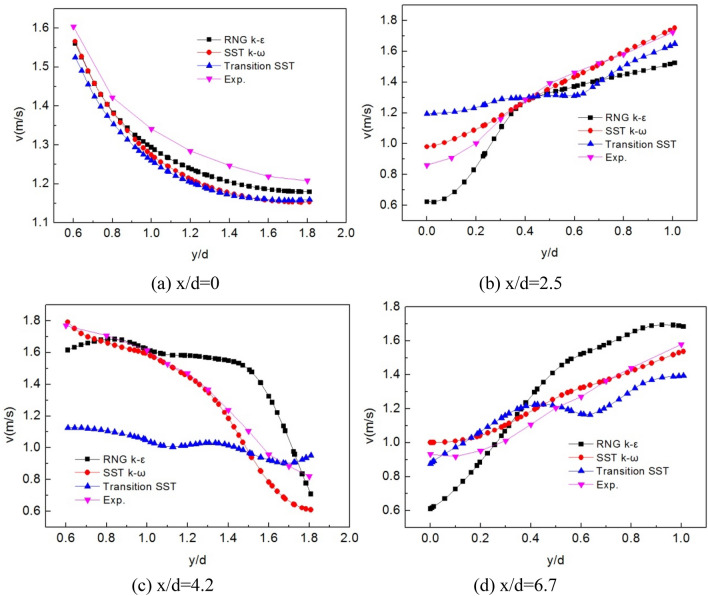
Comparison between model calculation and experiment.

The comparison of x velocities calculated by different turbulence models is shown in Fig. 5. Total of 4 positions, x/d = 0, 2.5, 4.2, and 6.7, are compared, respectively. Maximum relative error of different turbulence models are shown in Table 1. At the initial position, all three models agree well with the measured results. The transition SST has the most significant calculation error, but the maximum relative error is only 6.3%. At x/d = 2.5, the overall trend of the calculated results of the three models is consistent with the experimental result. Still, in numerical terms, the SST k–ω model is the most compatible with the experiment results, with a maximum relative error of 13.8%. At the same time, the RNG k–ε and the transition SST have the maximum relative error of 27.6% and 38.7%, respectively. With flow development, the difference between different models is more significant. At x/d = 4.2, the maximum relative error of transition SST is 36.3%. The SST k–ω is also the closest to the actual measurement value, with a maximum error of 16.2%. At x/d = 6.7, the maximum error of SST k–ω is 9.3%. According to the above results, the error of the SST k–ω model is the smallest, and it is in the best agreement with the measured results. Therefore, the SST k–ω model is adopted in this work.

**Table 1:** Maximum relative error of different turbulence models (%).

Positions	x/d = 0	x/d = 2.5	x/d = 4.2	x/d = 6.7
RNG k–ε	3.7	27.6	34.7	34.4
SST k–ω	5.6	13.8	16.2	9.3
Transition SST	6.3	38.7	36.3	11.7

The SST k–ω model was proposed by Menter^23^. Compared to most RANS models, the SST k–ω model provides better prediction of flow separation and performs well at lower pressure gradients, with high accuracy and economy. Different from standard k–ω, the SST k–ω model modifies the model constants and takes into account the transfer of turbulent shear stress, which uses k–ω in the inner region of the boundary layer and switches to k–ε in the free shear flow^24^.

k equation:

3 $$\frac{{\partial \left( {\rho k} \right)}}{\partial t} + \frac{{\partial (\rho u_{j} k)}}{{\partial x_{j} }} = P - \beta^{*} \rho \omega k + \frac{\partial }{{\partial x_{j} }}\left[ {\left( {\mu + \sigma_{k} \mu_{t} } \right)\frac{\partial k}{{\partial x_{j} }}} \right]$$

ω equation:

4 $$\frac{{\partial \left( {\rho \omega } \right)}}{\partial t} + \frac{{\partial (\rho u_{j} \omega )}}{{\partial x_{j} }} = \frac{\gamma }{{\nu_{t} }}P - \beta \rho \omega^{2} + \frac{\partial }{{\partial x_{j} }}\left[ {\left( {\mu + \sigma_{\omega } \mu_{t} } \right)\frac{\partial \omega }{{\partial x_{j} }}} \right] + 2\left( {1 - F_{1} } \right)\frac{{\rho \sigma_{\omega 2} }}{\omega }\frac{\partial k}{{\partial x_{j} }}\frac{\partial \omega }{{\partial x_{j} }}$$

where $P = \tau_{ij} \frac{{\partial u_{j} }}{{\partial x_{j} }}$, k is turbulence kinetic energy. ω is turbulence-specific dissipation rate. $\mu_{t}$ is turbulence viscosity coefficient.

5 $$\mu_{t} = \frac{{\rho a_{1} k}}{{\max \left( {a_{1} \omega ,\Omega F_{2} } \right)}}$$

*F*_1_ and *F*_2_ are mixed functions.

6 $$F_{1} = \tanh \left\{ {\left\{ {\min \left[ {\max \left( {\frac{\sqrt k }{{\beta^{*} \omega y}},\frac{500\nu }{{y^{2} \omega }}} \right),\frac{{4\rho \sigma_{\omega 2} k}}{{CD_{k\omega } y^{2} }}} \right]} \right\}^{4} } \right\}$$

7 $$F_{2} = \tanh \left\{ {\left[ {\max \left( {\frac{\sqrt k }{{\beta^{*} \omega y}},\frac{500\nu }{{y^{2} \omega }}} \right)} \right]^{2} } \right\}$$

According to Menter^23^, the coefficients for the model are shown in Table 2

**Table 2:** Coefficients for the SST k–ω model.

Coefficients	β*	*γ*	*β*	*σ*k	*σ*ω	*a*1
k–ω	0.09	0.556	0.0750	0.50	0.500	0.31
k–ε	0.09	0.440	0.0828	1.00	0.856	0.31

### Dynamics modeling of tube bundles vibration

The fluid-induced vibration of the tube bundles involves the interaction between the fluid and solid domain, which is challenging to calculate numerically^25^. Based on the computational fluid dynamics method, this paper establishes a simplified fluid-tube coupling vibration calculation model using the dynamic mesh method.

#### Simplification of vibration tube bundle structure model

**Figure 6:**
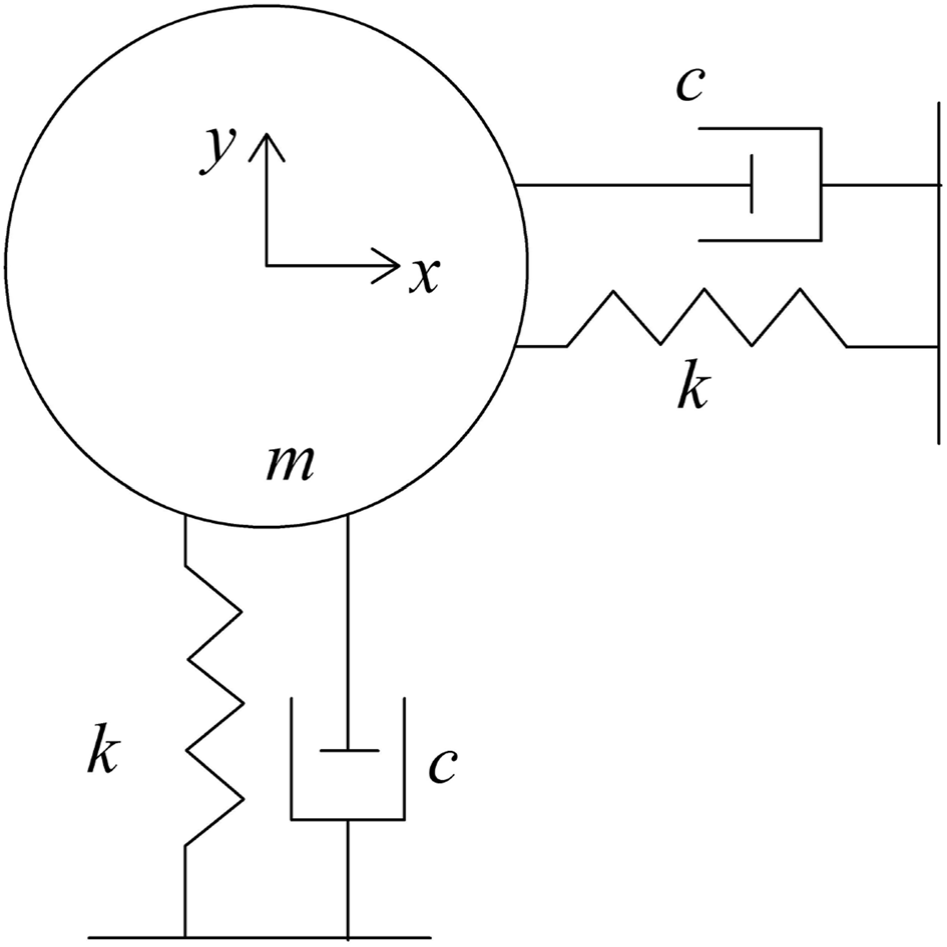
Simplified vibration flexible tube model (m—mass of the tube; k—stiffness of spring; c—damping of the spring-damp-mass system).

Because the calculation model that directly considers the coupling of tube bundle deformation and flow field change requires a lot of calculation and many iterations, the accuracy is difficult to guarantee. As shown in Fig. 6, the vibration model of the tube bundle is simplified into a two-dimensional spring-damp-mass system to improve the calculation efficiency^26,27^.

The stiffness, damping, and mass of the simplified tube bundles were calculated by referring to references^15,28,29^, respectively, and the values are shown in Table 3.

**Table 3:** The mass, damping, and stiffness.

m	c	k
2.06 kg	4 Ns/m	97,003 N/m

#### Solution of rigid body motion of bundle

The fluid force on the tube bundle is obtained by solving the fluid motion equation. Taking the fluid force as known, the rigid body motion equation on the tube bundle can be solved, and the fluid–structure coupling of the tube bundle system is realized. The rigid body's motion equation at the bundle's boundary is solved by the Newmark-β integral method^30,31^.

The vibration differential equation of the tube is

8 $$m\ddot{x} + c\dot{x} + kx = F$$

where m, c, k, and F are mass, damping, stiffness of the spring-damp-mass system, and fluid force applied to the tube bundles. $x$, $\dot{x}$, $\ddot{x}$ are the displacement, velocity and acceleration of tube^32,33^.

According to the Newmark-β method,

9 $$\dot{x}_{t + \Delta t} = \dot{x}_{t} + \left[ {\left( {1 - \beta } \right)\ddot{x}_{t} + \beta \dot{x}_{t + \Delta t} } \right]\Delta t$$

10 $$x_{t + \Delta t} = x_{t} + \dot{x}_{t} \Delta t + \left[ {\left( {0.5 - \gamma } \right)\ddot{x}_{t} + \gamma \ddot{x}_{t + \Delta t} } \right]\Delta t^{2}$$

where $x_{t}$, $\dot{x}_{t}$ and $\ddot{x}_{t}$ are the displacement, velocity, and acceleration at t. $x_{t + \Delta t}$, $\dot{x}_{t + \Delta t}$ and $\ddot{x}_{t + \Delta t}$ are the displacement, velocity and acceleration at *t* + Δ*t*, $\gamma$ and $\beta$ are coefficient of Newmark-β method. Δ*t* is the time step.

According to Eqs. (9) and (10),

11 $$\ddot{x}_{t + \Delta t} = \frac{1}{{\gamma \Delta t^{2} }}\left( {x_{t + \Delta t} - x_{t} } \right) - \frac{1}{\gamma \Delta t}\dot{x}_{t} - \left( {\frac{1}{2\gamma } - 1} \right)\ddot{x}_{t}$$

12 $$\dot{x}_{t + \Delta t} = \frac{\beta }{\gamma \Delta t}\left( {x_{t + \Delta t} - x_{t} } \right) - \left( {1 - \frac{\beta }{\gamma }} \right)\dot{x}_{t} - \left( {1 - \frac{\beta }{2\gamma }} \right)\ddot{x}_{t}$$

Then, the vibration differential equation of the system at time t + Δt is

13 $$m\ddot{x}_{t + \Delta t} + c\dot{x}_{t + \Delta t} + kx_{t + \Delta t} = F$$

$x_{t + \Delta t}$ can be expressed as $K^{*} x_{t + \Delta t} = F_{t + \Delta t}^{*}$.

Where

14 $$K^{*} = k + \frac{1}{{\gamma \Delta t^{2} }}m + \frac{\beta }{\gamma \Delta t}c$$

15 $$\begin{aligned} F_{{t + \Delta t}}^{*} = & F_{{t + \Delta t}} + m\left[ {\frac{1}{{\gamma \Delta t^{2} }}x_{t} + \frac{1}{{\gamma \Delta t}}\dot{x}_{t} + \left( {\frac{1}{{2\alpha }} - 1} \right)\ddot{x}_{t} } \right] \\ & + c\left[ {\frac{\beta }{{\gamma \Delta t}}x_{t} + \left( {\frac{\beta }{\gamma } - 1} \right)\dot{x}_{t} + \left( {\frac{\beta }{{2\gamma }} - 1} \right)\Delta t\ddot{x}_{t} } \right] \\ \end{aligned}$$

where $K^{*}$ is effective stiffness. $F_{t + \Delta t}^{*}$ is effective load.

When the mass, stiffness, and damping of the vibrating tube bundle are obtained, the dynamic response of the next time can be obtained by substituting the previous time's dynamic response into the system's initial state.

### Calculation model for fluid-induced vibration of different stiffness tube

#### Calculation domain and model

Due to the large number of tubes in the heat exchanger, the cost of numerical calculation is significant, making it unrealistic to model and study the vibration of all tubes. It makes sense to reduce the number of tubes through reasonable simplification^34,35^. The three-dimensional model of the tube bundle is simplified into a two-dimensional model. At the same time, only a dozen tubes in typical positions are selected for targeted research, and the calculation amount and time are reduced under the premise of accuracy.

**Figure 7:**
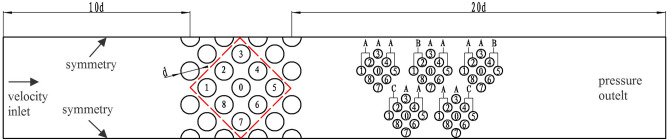
Physical model of vibration of different stiffness tube bundles.

The geometric structure of the numerical calculation model is shown in Fig. 7. Take the corner square arrangement tube bundle as the research object. The heat exchange tube is simplified into a bundle with seven rows and columns. Fluid flows in from the left side and out from the right, and the top and bottom sides are symmetric. The distance from the inlet to the first tube bundle is ten times the tube bundle diameter, and from the last tube bundle to the outlet is 20 times the tube diameter. The tube bundle has a diameter of 25 mm and a pitch ratio of 1.28.

Figure 7 also gives the scheme of the tube bundle stiffness combination. Tube 0 is the target tube, and tubes 1–8 are flexible tubes, which have been marked in the dotted box. The calculation method is the same as the target tube, and self-excited vibration can occur. The unlabeled tubes outside the dotted box are rigid, assuming no vibration occurs in the flow field and no particular definition is required. Three kinds of stiffness were considered: large, normal, and small. Large stiffness was taken as two times the normal stiffness, and the small stiffness was 1/2 of the normal stiffness. In the calculation, only the influence of stiffness change is considered. Thus, the mass of the tube bundle remains unchanged, and the inherent frequency changes due to the stiffness change. The stiffness and corresponding inherent frequency are shown in Table 4.

**Table 4:** Stiffness and inherent frequency of tube.

Different stiffness	Normal stiffness	Large stiffness	Small stiffness
Symbol	A	B	C
Stiffness (N/m)	97,003	194,006	48,501.5
Inherent frequency (Hz)	38	53	26

The stiffness of tube 0 is kept unchanged, and the tube bundles' stiffness is changed upstream or downstream of the target tube. The actual structure of the heat exchanger is referred to ensure that there are only two stiffnesses in the flexible tube bundle. The upstream and downstream are not repeated to determine the stiffness combination scheme.

#### Computational mesh and mesh independence study

**Figure 8:**
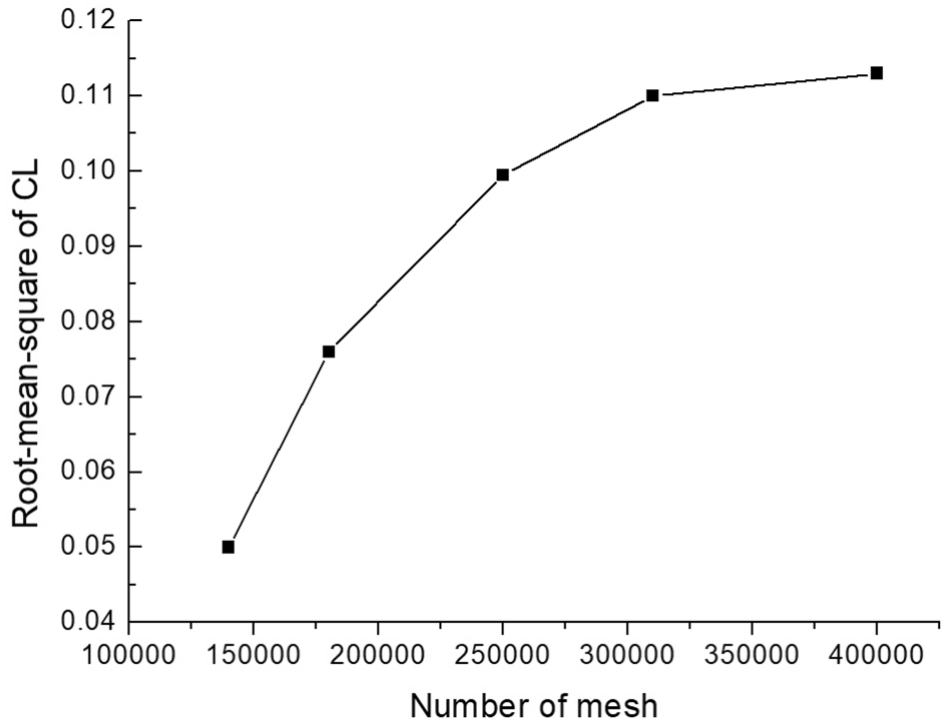
Grid independence verification.

Mesh independence study is very important for the numerical study authenticity. Based on the number of mesh cells, different mesh densities are used to find a compromise between computational cost and accuracy. Numerical calculations were performed on meshes of 140,000, 180,000, 250,000, 310,000 and 400,000 (corresponding to mesh spacings of 1 mm, 0.8 mm, 0.6 mm, 0.5 mm and 0.4 mm) with inlet velocity of 0.5 m/s. To simplify the calculations, all tube bundles are set to be rigid. The lift coefficients of the target tube (tube 0) are compared under different meshes to determine the appropriate mesh size. The results are shown in Fig. 8. After the number of meshes reaches 310,000, as shown in Fig. 8, the calculation results are almost unaffected by the sparsity of the grids. Relative error between number of 310,000 and 400,000 is just 2.7%. So this paper's research is conducted using 310,000 grid cells.

**Figure 9:**
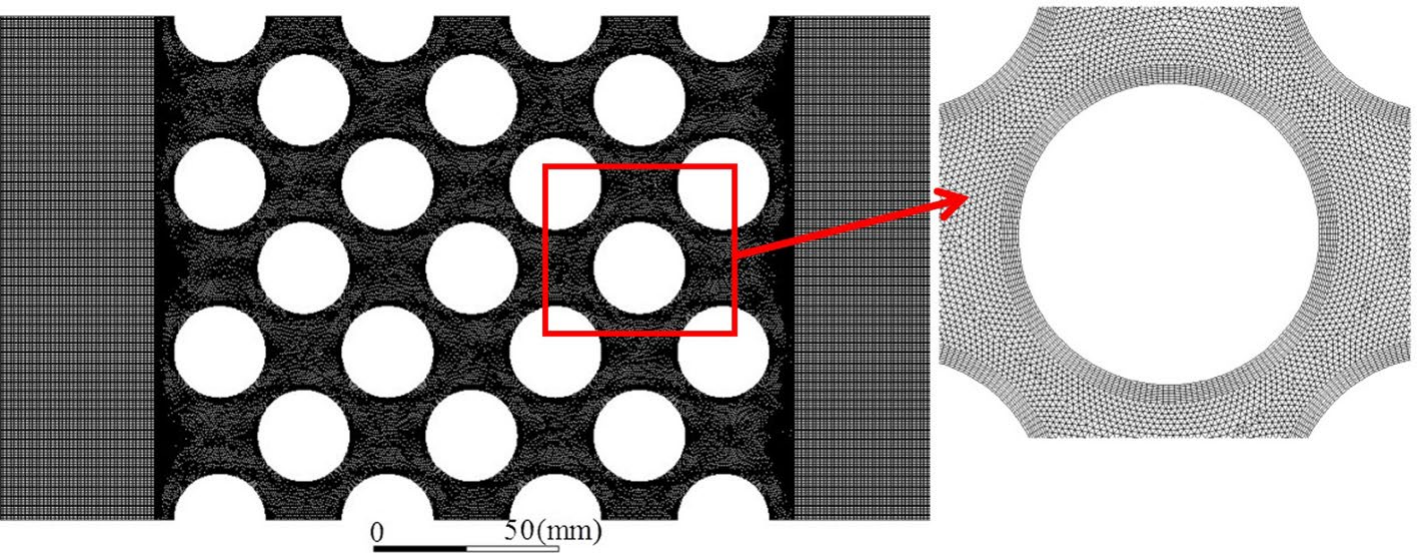
Computational mesh.

The computational mesh is shown in Fig. 9. Due to the calculation requirements of the dynamic mesh, the combining structure and unstructured mesh is used, in which an unstructured mesh was used in the tube bundles gaps. The near tube wall zones were divided into a structure mesh. The wall surface of the bundle was encrypted. The number of encryption layers was 8, the initial height was 0.2 mm, and the mesh growth rate was 1.1. The bundle's front and rear flow areas adopt a quadrilateral mesh structure.

#### Boundary condition and solution settings

Because of the need to simultaneously calculate the vibration characteristics of the tube bundle and realize coupling with the fluid, it is necessary to perform transient calculation^36^. The boundary condition and solution Settings are shown in Table 5.

**Table 5:** Calculation boundary and solution settings.

Setting	Details
Calculation type	Transient analysis
Fluid	Water
Turbulence model	SST k–ω
Arithmetic	SIMPLE
Inlet condition	Velocity inlet (0.1–0.4 m/s)
Outlet condition	Pressure outlet (0.1 MPa)
Residual	10^–5^
Time step	0.0001 s

## Experimental validation of computational models

### Experimental facility

**Figure 10:**
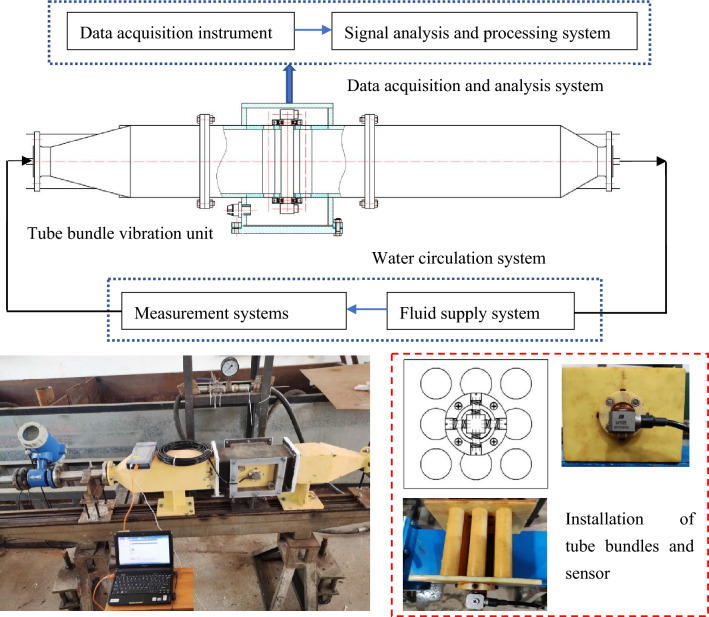
Experimental facility.

A vibration experimental facility for a single flexible tube bundle is established to validate the fluid–structure interaction computational model based on dynamic mesh and linear solution methods. The experimental facility is shown in Fig. 10. It consists of three parts: a water circulation system, a data acquisition and analysis system, and the tube bundle vibration unit. The water circulation system comprises a pump, a supply adjustment system, flow and pressure measuring devices, a water tank, and pipe. Within the tube bundle vibration testing unit, the internal space measures 130 mm × 130 mm in cross-section. The experimental tube bundle is arranged in a 3 × 3 square configuration, with a flexible tube at the center, while the other tubes are firmly connected to the tube plate. Springs are employed to constrain the middle tube bundle to the fixed tube plate. Eight springs are evenly distributed at four positions—horizontally and vertically—at both ends of the flexible tube. Sensors can be fixed and connected through bolts at the ends of the central flexible tube. The data acquisition and analysis system is the DH5981 data acquisition system, using the complementary DHDAS software for signal analysis and processing. The sensors utilized are IEPE underwater piezoelectric acceleration sensors, with their relevant parameters detailed in Table 6.

**Table 6:** Technical parameters of acceleration sensor.

Dynamic indicators		Electrical parameter	
Axial sensitivity	10 mV/m s^−2^	Constant current source voltage	18–30 V_DC_
Measuring range	± 50 g	Operating constant current	2–20 mA
Linear	< 1%	Output signal	> 5 V_P_
Maximum transverse sensitivity	< 5%	Output impedance	< 100 Ω
Frequency response	0.5–10000 Hz	Operating temperature	− 40 to 80 °C
Resolution	0.0002 g		

### The comparison between numerical computational results and experimental data

**Figure 11:**
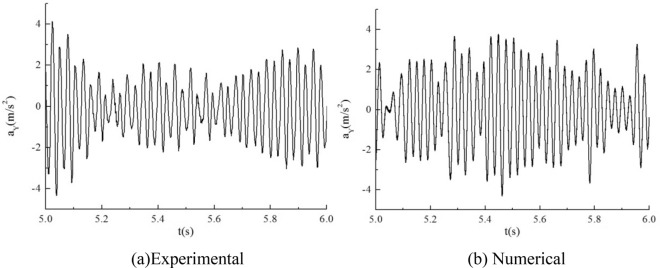
Time course curve of acceleration.

**Figure 12:**
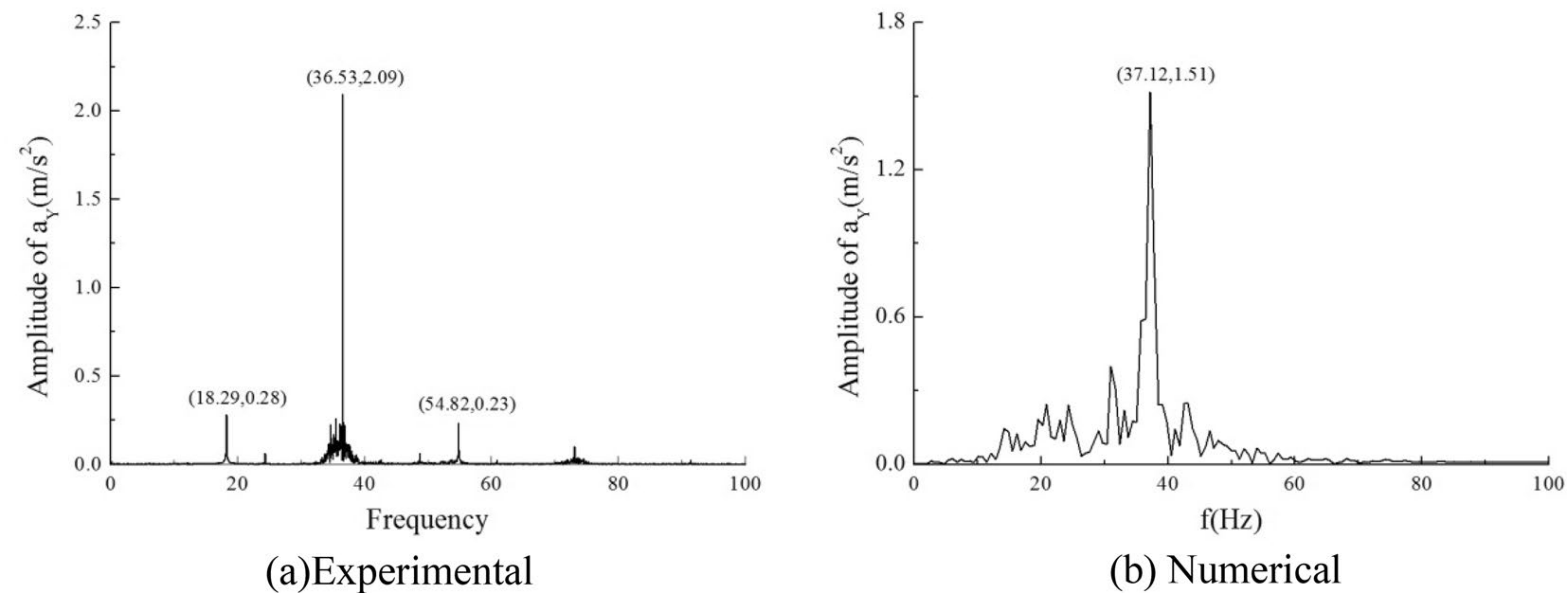
Spectrogram of acceleration.

In Figs. 11 and 12, the acceleration time course curves and spectral diagrams for both experimental and numerical simulations are presented under the condition of an inlet flow velocity of 0.1 m/s. It can be observed that, overall, the trends of the two are quite similar, with the simulated values slightly lower in magnitude compared to the experimental values. In the frequency spectrum, the main frequencies of the two are also relatively close.

## Analysis of coupling vibration characteristics of tube bundles

### Vorticity distribution between tube bundles

**Figure 13:**
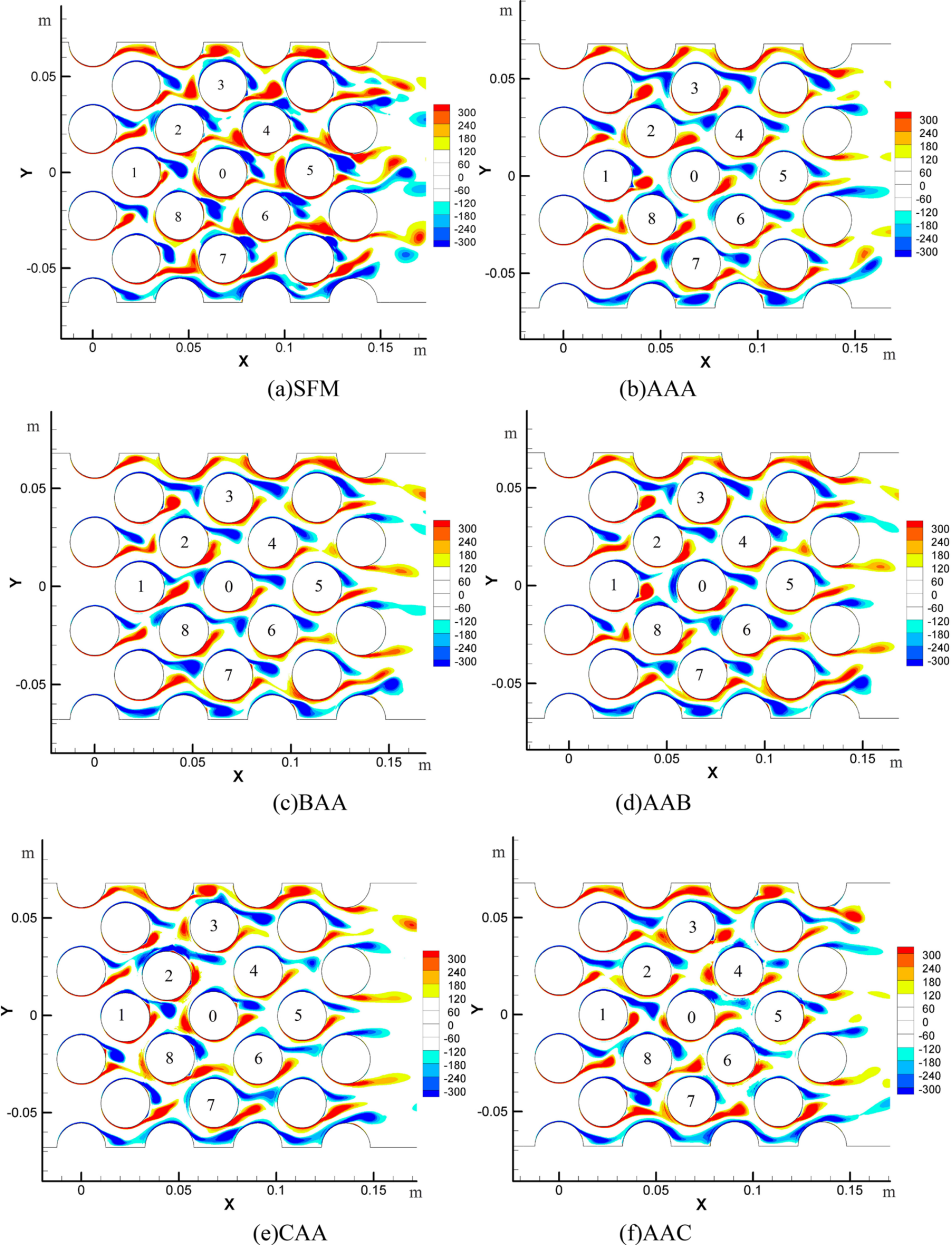
Vorticity of tube bundle with different stiffness combinations; (**a**) SFM; (**b**) AAA; (**c**) BAA; (**d**) AAB; (**e**) CAA; (**f**) AAC.

Figure 13 shows the vorticity distribution between multiple flexibly-mounted tubes with different stiffness. SFM represents a single flexible mounted tube bundle with A target tube stiffness. The meanings of other labels are shown in Fig. 7. The vorticity distribution shows the process of vortex formation, shedding, and adhesion between the tube bundles^37^. The first tube bundles generate the vortices, which shed, attach, and move downstream along the flow direction. Due to the interaction between the tightly packed tube bundles, the vortices between the bundles could not fully develop until the last row of tube bundles. The vortices with different shedding characteristics fell off and formed a complete Karman vortex street^38^.

When the flexible tubes are added around the target tube, the vortex shape remains unchanged, the vortex separation point is more significant than 120°, and the vortex width is narrow. In general, the overall vortex strength of the multi-flexible tube bundles is lower than the single flexibly-mounted tube bundles, so the pressure fluctuation caused by the vortex shedding will also decrease. The vortex shedding patterns on the nine flexible mounted tubes are basically the same for AAA, BAA, and AAB. The influence of the upstream tube on vortex generation and shedding of the downstream tube is weakened, and the parallel vortex on the target tube is less than that on a single flexible mounted tube. When the upstream or downstream are large stiffness tubes, the vibration law of the target tube tends to a single flexible mounted tube because the vibration amplitude of a large stiffness tube decreases.

As a comparison, the upstream or dowstream are small stiffness for CAA and AAC. Under the same condition, the amplitude of the small stiffness tube is larger. The displacement of tube 1 in the lift direction is large, resulting in a certain distance between tube 1 and downstream tube 0 in the lift direction. When the red vortex at the lower part of tube 1 falls off and is attached to tube 0 again, it is already below the former freeze point of tube 0, and the pressure at the lower part of tube 0 increases. Meanwhile, the blue vortex at the upper part of tube 0 is in the shedding stage, and the upper pressure further decreases.

Combining the two causes a larger amplitude of the target tube 0. For AAC, when the low stiffness tube is added downstream, the large vibration of 4 or 6 tubes prevents the shedding of the vortex of 0 tubes to some extent, the pressure release is blocked, and the surface force of 0 tubes is increased. When the vortices above and below the central target tube finish shedding, it is a complete vortex-shedding cycle. It is obtained that the vortex shedding frequency between tubes is about 25 Hz, which is the dominant frequency of the flow field.

### Time–frequency analysis of tube bundle vibration

**Figure 14:**
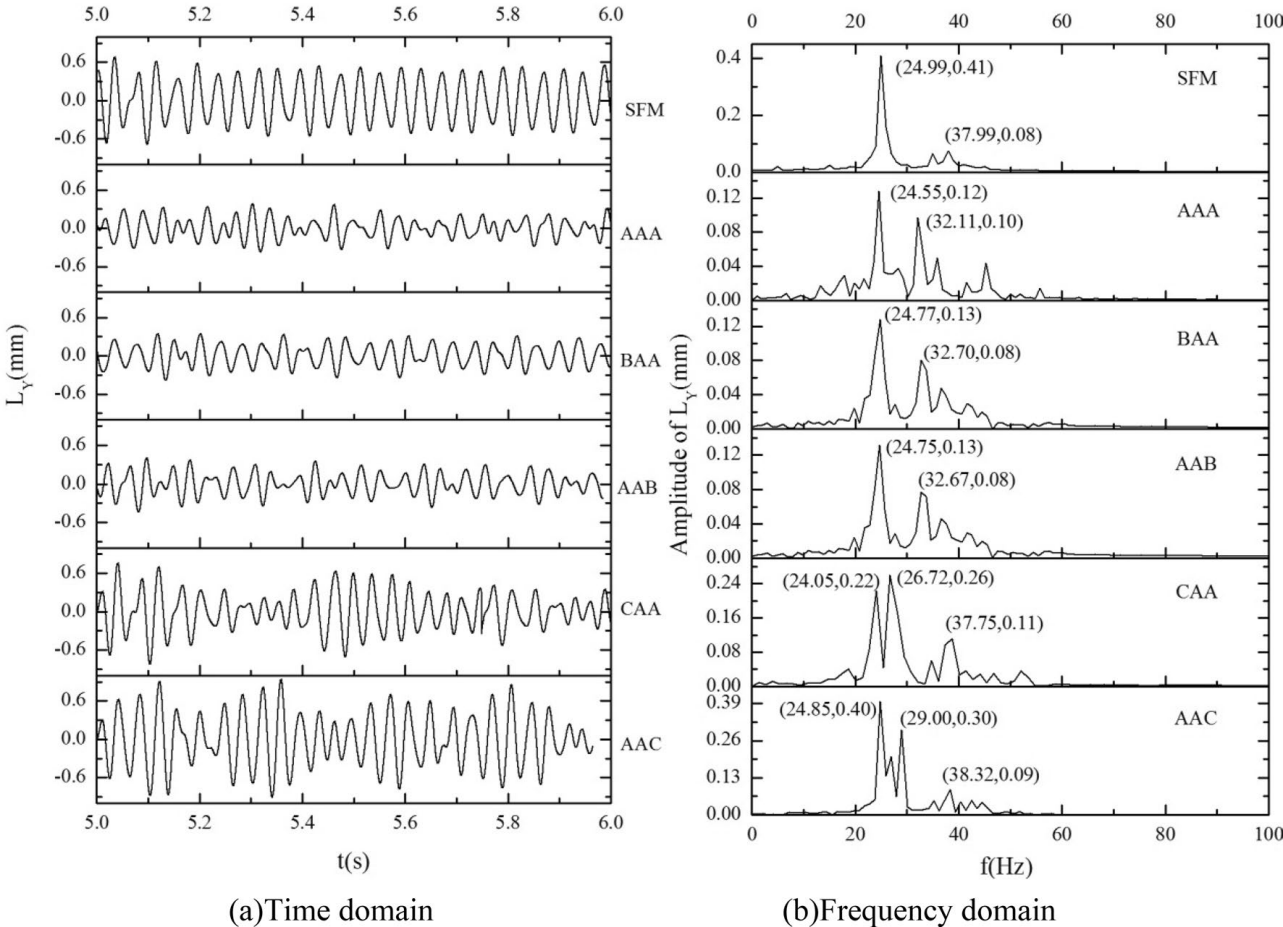
Amplitude time–frequency analysis of target tube under different stiffness combinations; (**a**) time domain; (**b**) frequency domain.

The amplitude of target tube 0 is analyzed by time–frequency. The result is shown in Fig. 14. From the amplitude time history curve, and it can be found that compared with the single flexible mounted tube, the amplitude of the target tube is smaller under the equal stiffness AAA, the upstream or downstream large stiffness BAA or AAB. The amplitude of the target tube is significantly suppressed. It should be noted that the upstream and downstream tube bundles are moderately stimulated under this working condition, and the vibration amplitude and phase of the upstream and downstream tubes are in a suitable range, which produces a good vibration suppression effect. For CAA and AAC, the amplitude of the target tube is equal to or larger than that of the single flexible tube, which can be considered to be caused by the flow block and vortex detachment deviation caused by the large motion of the low-stiffness tube. In conjunction with Table 7, it can be seen that the amplitude of AAA is the smallest, and its root mean square amplitude is reduced by more than 50% compared with a single flexible tube. The amplitude of BAA and AAB is about 10% higher than AAA's. The amplitude of the target tube is larger for CAA and AAC, with small stiffness.

**Table 7:** Amplitude of target tube under different stiffness combination schemes.

Stiffness combination scheme	SFM	AAA	BAA	AAB	CAA	AAC
Maximum amplitude (mm)	0.686	0.352	0.383	0.405	0.767	0.941
Root-mean-square amplitude (mm)	0.324	0.155	0.173	0.167	0.295	0.413

From the spectrum diagram, we can find that the dominant frequency of the vibration of the target tube is about 25, which is the vortex-shedding frequency of the flow field. The addition of upstream and downstream flexible tubes complicates the target tube's amplitude frequency^39^. In addition to the primary frequency, there are multiple peaks, which should include the inherent frequency of the bundle and the exciting frequency of turbulence.

### Trajectories of each flexible mounted tube under different stiffness combinations

**Figure 15:**
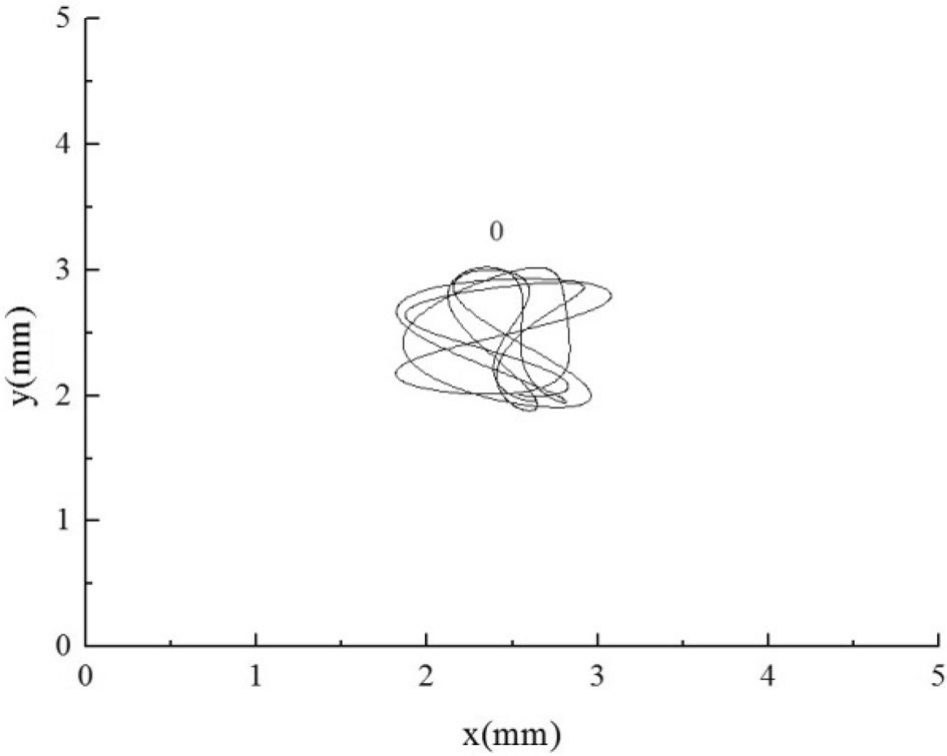
Tube vibration trajectories of SFM.

**Figure 16:**
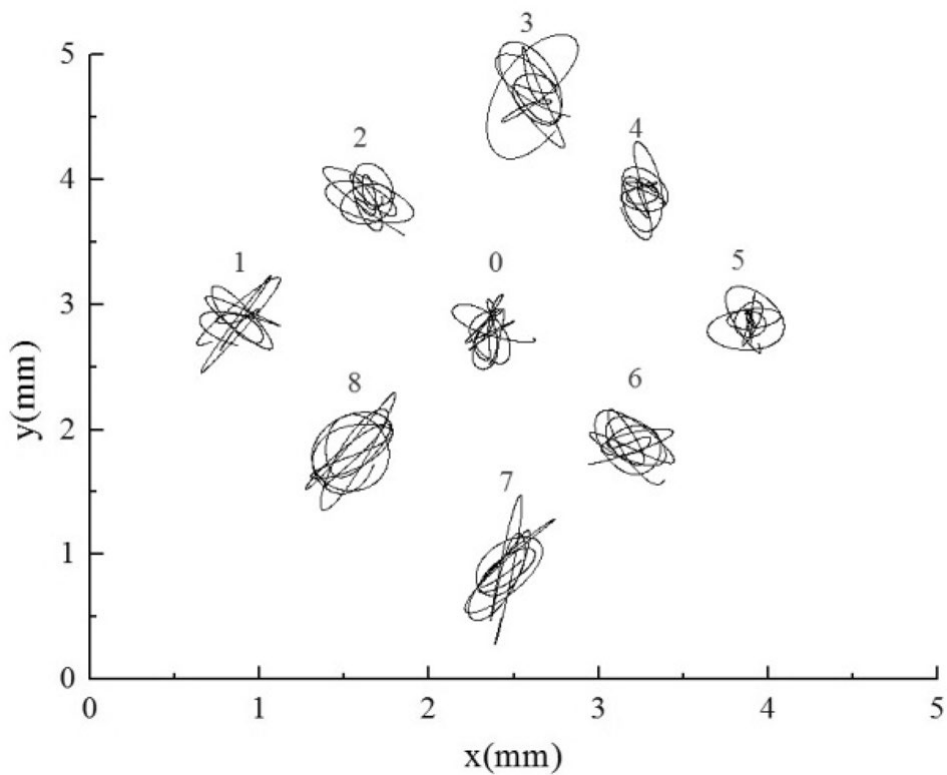
Tube vibration trajectories of AAA.

**Figure 17:**
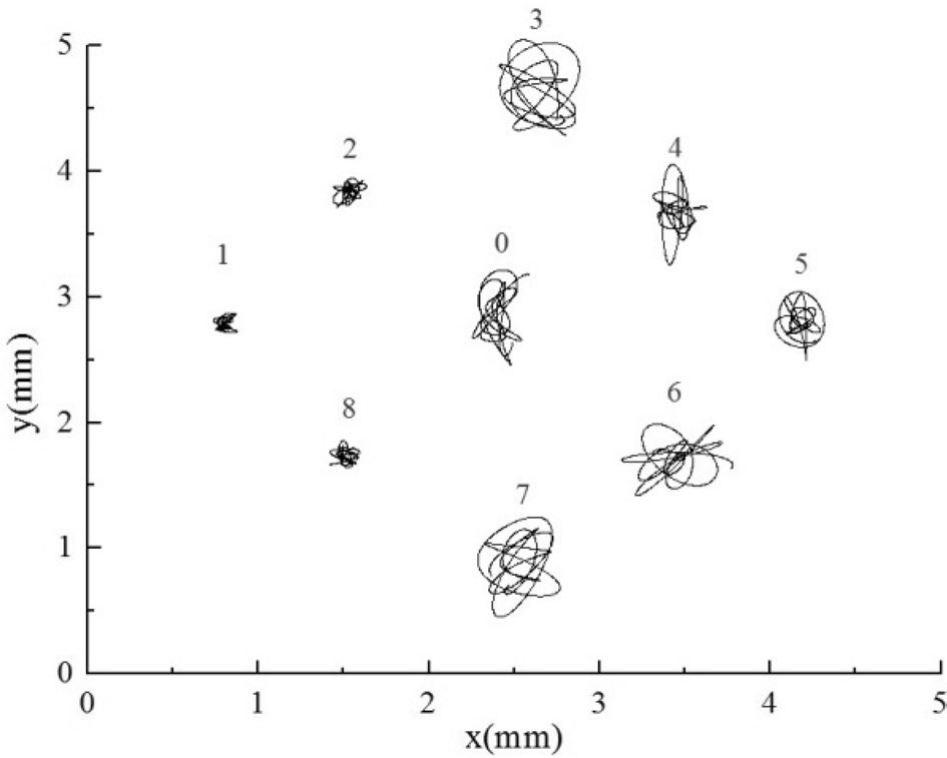
Tube vibration trajectories of BAA.

**Figure 18:**
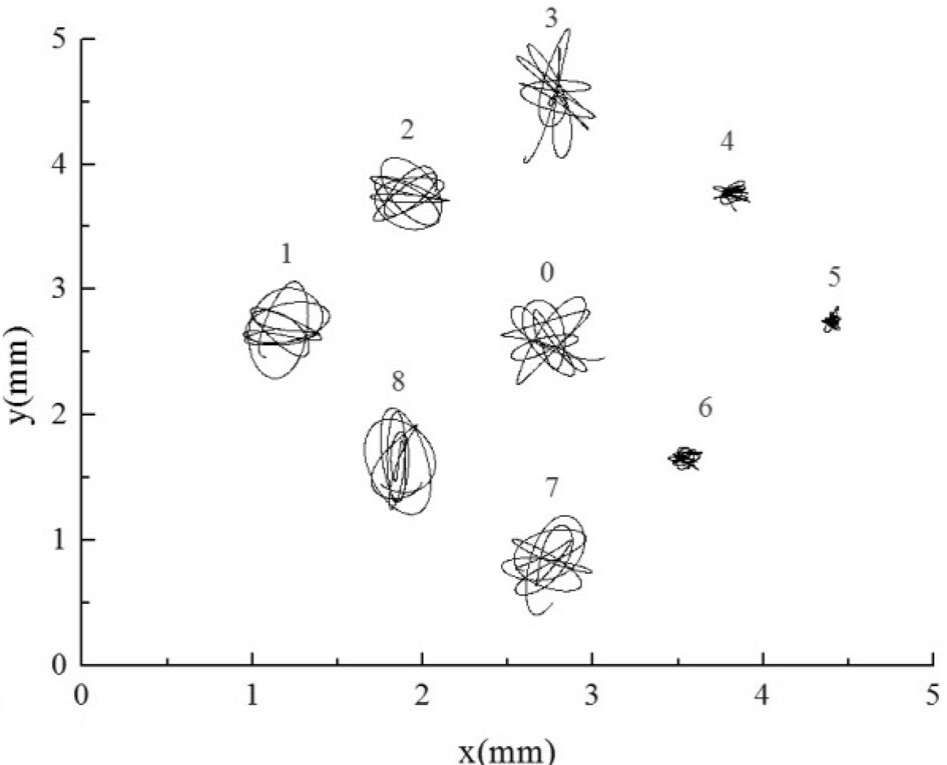
Tube vibration trajectories of AAB.

**Figure 19:**
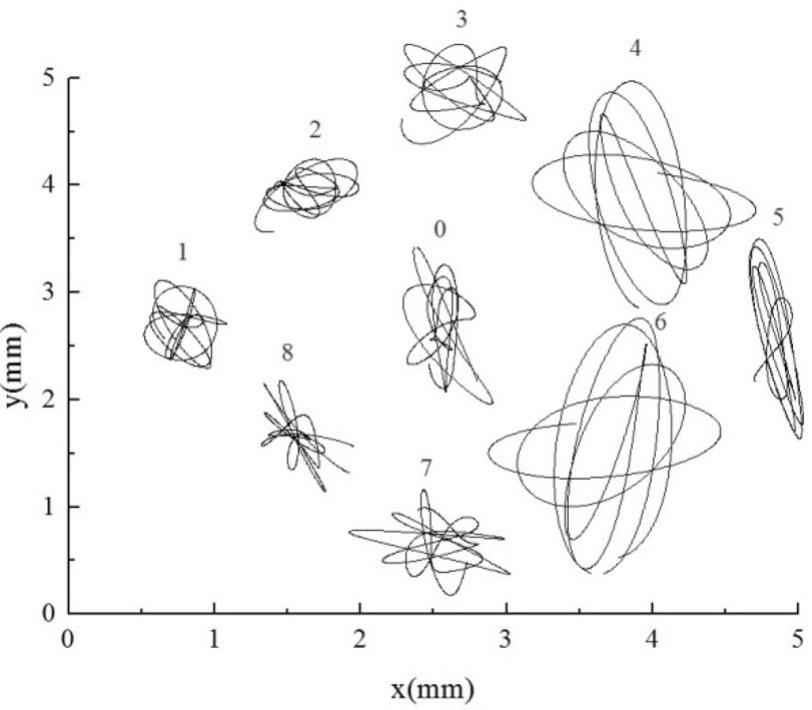
Tube vibration trajectories of CAA.

**Figure 20:**
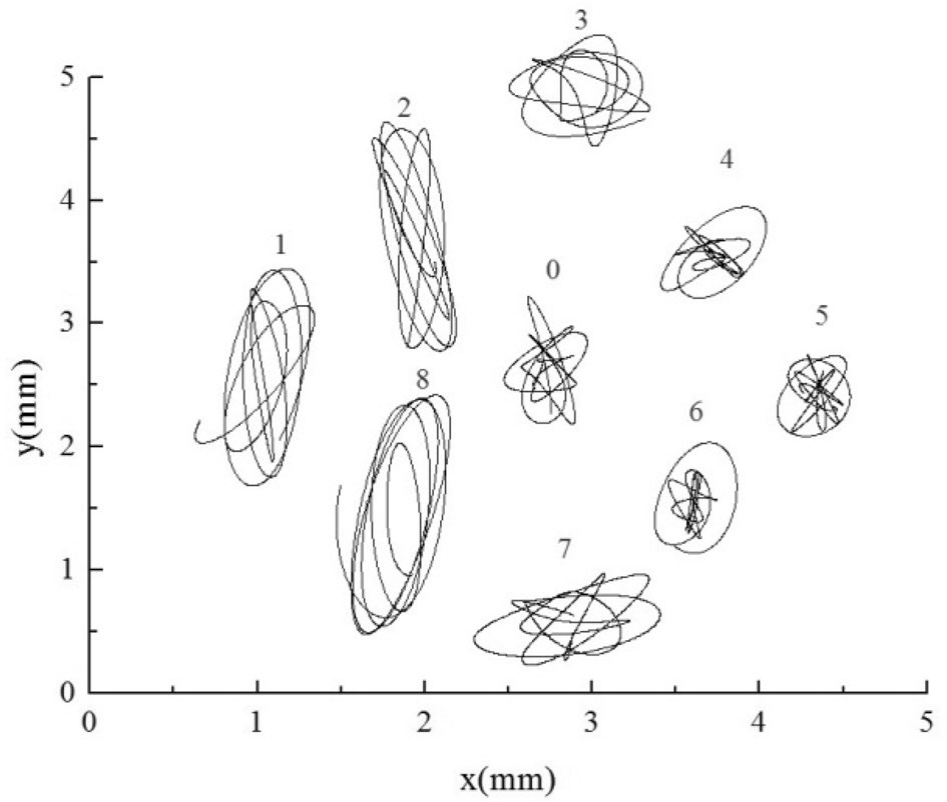
Tube vibration trajectories of AAC.

The motion trajectories of all flexible tubes were analyzed, and the motion trajectories of tube bundles within 0.3 s were selected, as shown in Figs. 15, 16, 17, 18, 19 and 20. For the equal stiffness AAA, each tube presents a typical eight or elliptic vibration trajectory^40^, and the amplitude of the tube bundle in the lift direction is slightly larger than that in the drag direction. The tube bundles move approximately periodically. Among them, the amplitudes of the upstream tube bundles 1, 2, 3, 7, and 8 are slightly larger than that of the downstream tube bundles 4, 5, and 6. The front of the upstream tube bundles are direct flow or fixed tubes, and the coupling characteristics between other tubes and a single flexible tube have similarities, so the amplitude is more prominent. The front flexible tubes affect the downstream tube bundles, and the vibration is suppressed.

For BAA, as shown in Fig. 17, the upstream tubes 1, 2, and 8 are large stiffness tubes. Under the premise of no resonance, the amplitude of large stiffness tubes is significantly lower than that of small stiffness tubes, as reflected in the trajectory. The amplitude of large stiffness tubes is one order of magnitude smaller than that of normal stiffness tubes. According to the trajectory, the amplitude of the target tube and other normal stiffness tube bundles downstream did not decrease after adding the upstream large stiffness tube, and the amplitude of the lift direction was more significant than the resistance direction.

The downstream tubes 4, 5, and 6 for AAB are large stiffness tubes. As shown in Fig. 18, the amplitude of the large stiffness tube is significantly lower than that of the normal stiffness tube, regardless of whether it is upstream or downstream. Adding the downstream tubes with large stiffness increases the amplitude of the target tube slightly. The influence of stiffness change of the downstream tube bundle on the target tube is more potent than that of the upstream tube bundle.

As shown in Fig. 19, when small stiffness tubes are added upstream, all flexible tube bundles amplify. Among them, the displacement of small stiffness tubes 1, 2, and 8 in the lift direction is about an order of magnitude larger than that of normal stiffness tubes, and the maximum amplitude on one side is greater than 2 mm, that is, more than 0.08 days. The three tubes with this stiffness are already in resonance from the trajectory. Because of the fluid excitation force generated by the larger amplitude of the upstream small stiffness tubes, the vibration of the downstream normal stiffness tubes is also enhanced, and the increase of the 3 and 7 tubes is the largest.

The AAC is a downstream small stiffness tube bundle. As shown in Fig. 20, under this condition, the small stiffness tubes 4, 5, and 6 have large amplitudes, and the maximum amplitude on one side is more significant than 2.3 mm, which is about 0.1 days. These tubes should be in a resonance state. The trajectories of the three small-stiffness tubes are also slightly different. Tube 5 shows noticeable vibration enhancement in the lift direction. However, the resistance direction is the same as that of the normal stiffness tubes, and the obstruction of the Tube 4 and the Tube 6 in the flow direction. Because of the influence of the small stiffness tube downstream, the vibration of the normal stiffness tube bundle upstream is enhanced.

### Interactions between pipes at different flow rates.

**Figure 21:**
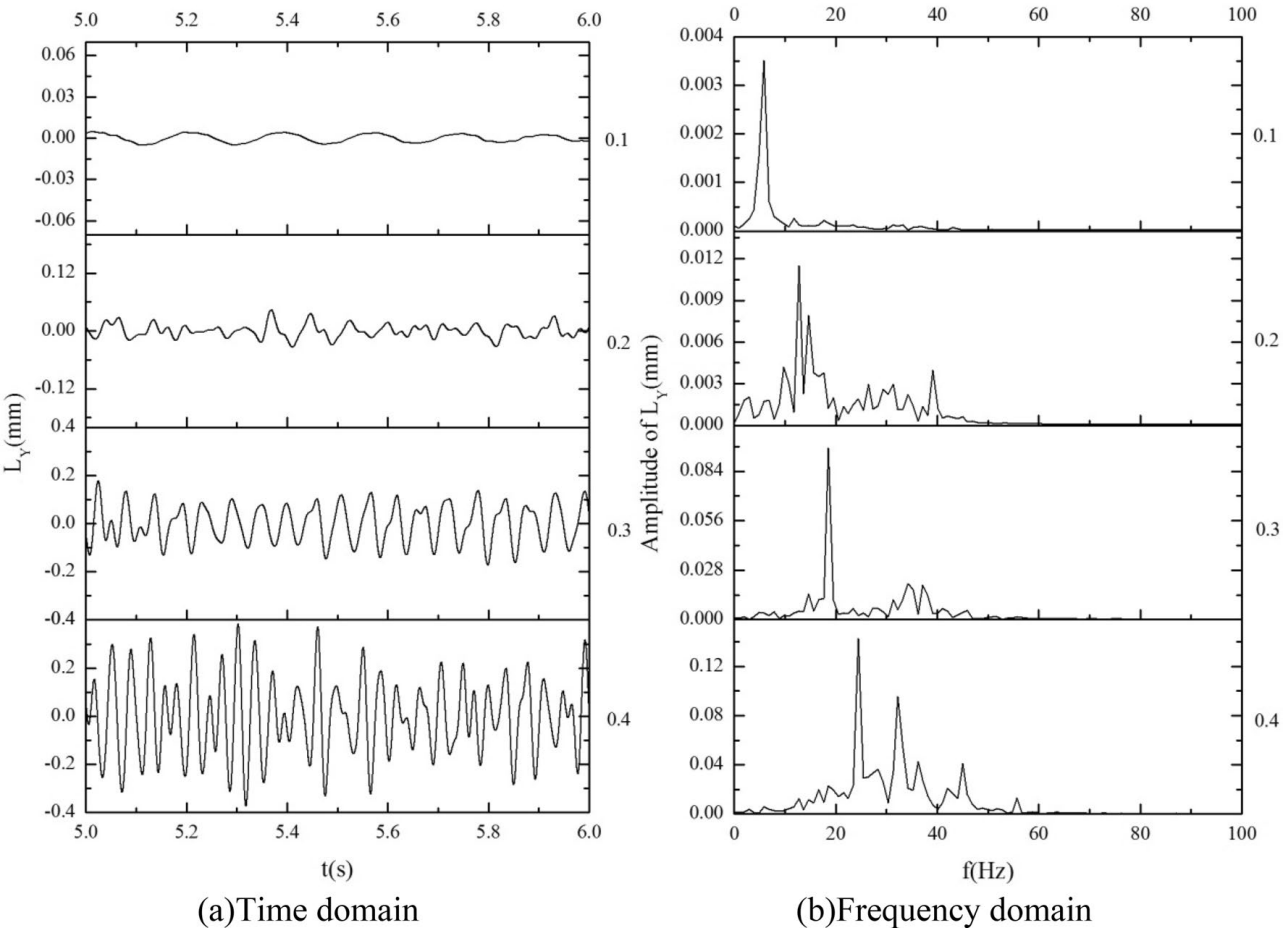
Amplitude time–frequency analysis of target tube under different stiffness combinations; (**a**) time domain; (**b**) frequency domain.

The vibration response of the AAA combination is analyzed at different flow velocities, and the amplitude-time curves and frequency-domain curves are presented in Fig. 21. The inlet velocity range is 0.1–0.4 m/s.

As the flow velocity increases, the dominant frequency of the flow field also increases, showing a basic consistency with the single-flexible tube under the same flow conditions. At lower flow velocities, the vibration frequency of the target tube is relatively singular, exhibiting only one dominant frequency throughout the entire time domain, which is the dominant frequency of the flow field.

The amplitude and dominant frequency of the vibration of target tube 0 both increase with the increase in flow velocity. Additionally, non-dominant frequency components in the spectrum also increase, including both the inherent frequencies of the tube bundle and indistinct turbulent disturbance components. This suggests that the increase in flow velocity enhances the coupling vibration effects between the tube bundles and the degree of turbulence-induced vibration.

**Figure 22:**
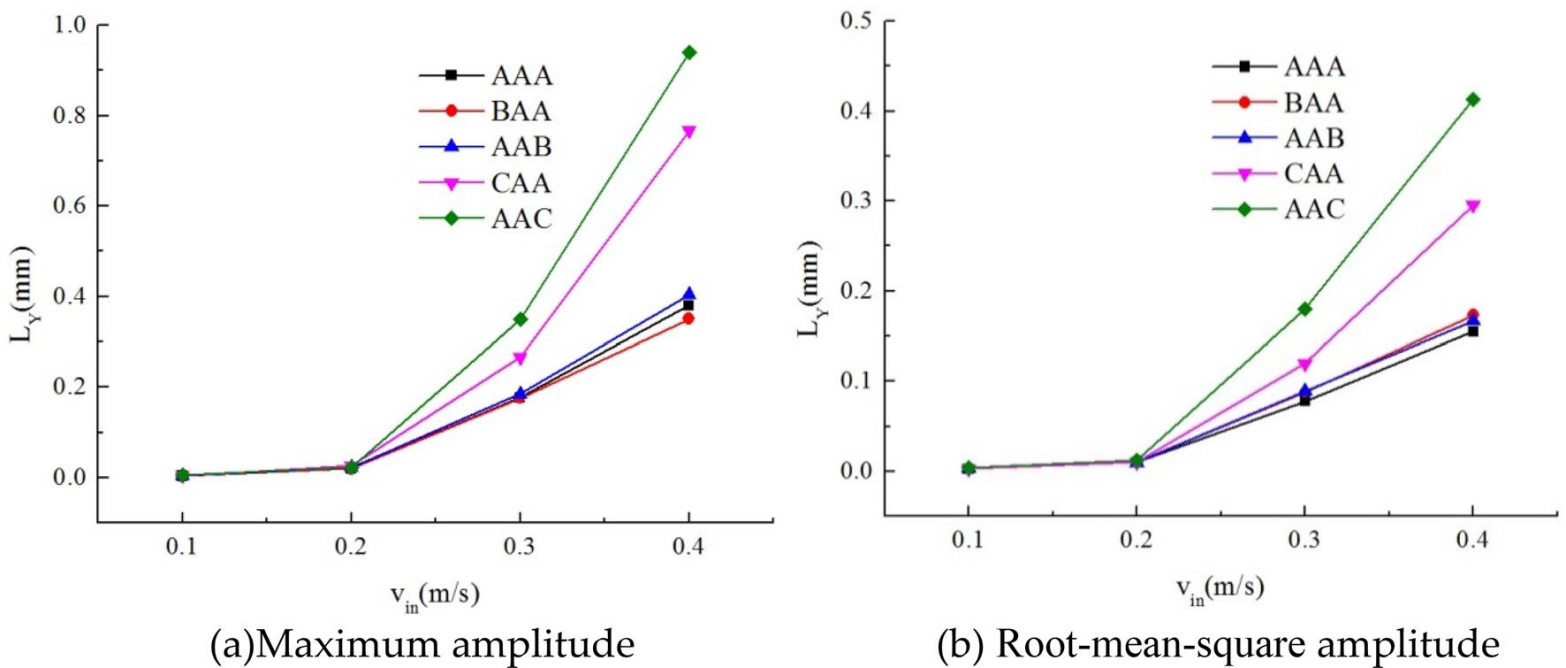
Maximum amplitude and root-mean-square amplitude of target tube 0; (**a**) maximum amplitude; (**b**) root-mean-square amplitude.

Figure 22 illustrates the maximum amplitude and root-mean-square amplitude of target tube 0 concerning various stiffness combinations at different incoming flow velocities. Under AAA, AAB or BAA, the target tube's amplitude notably decreases compared to configurations with lower stiffness in the upstream or downstream sections. Particularly, the upstream higher stiffness configuration consistently demonstrates a significant dampening effect on the downstream target tube across all flow velocities. Once the incoming flow velocity surpasses 0.2 m/s, there is a noticeable sharp increase in the amplitude of lower stiffness tube combinations, either upstream or downstream. This trend aligns with the characteristic behavior of fluid-elastic instability within tube bundles. The presence of lower stiffness tube combinations, whether upstream or downstream, to some extent, diminishes the critical velocity required for fluid-elastic instability, rendering the tube bundle more susceptible to instability.

## Conclusions

The numerical calculation of coupling vibration of heat exchange tube bundles was completed using dynamic grid technology with rigid body motion equation. The vortex and vibration characteristics of tube bundles were analyzed, and the following conclusions were obtained:

- Vortices shedding on the tube bundles are the primary cause of flow field fluctuation, which is the root cause of tube bundle vibration. The vortex shedding frequency on the bundles is the dominant frequency of the flow field. The tube bundles take the flow field dominant frequency as the primary vibration frequency and include the tube bundle's inherent frequency.
- The vibration of multi-flexible tube bundles changes the vortex shedding distribution between tube bundles, and the vibration of the target tube differs from that of the single flexible tube bundle. On the premise that no resonance occurs, the upstream and downstream flexible tubes can suppress the vibration of the target tube to a certain extent, resulting in lower amplitude and more complex vibration frequency. However, the dominant frequency is the vortex shedding frequency.
- The stiffness changes of the upstream and downstream tube bundles will affect the vibration of the central target tube. On the other hand, within the calculated range, no matter whether increasing the stiffness or decreasing the stiffness, the vibration of the central target tube cannot be better suppressed, and the amplitude of the central target tube is the smallest when the stiffness of the upstream and downstream and the target tube is the same. Reducing the stiffness of the tube bundles upstream or downstream will, to some extent, decrease the critical flow velocity of the target tube, making it more prone to experiencing fluid-elastic instability. This work can be used for better design and vibration suppression of shell-and-tube heat exchangers.

## Data Availability

The data that support the findings of this study are available from the corresponding author upon reasonable request.

## References

[1] Kumar A, Bhattacharya M (2016). Numerical analysis of aseptic processing of a non-newtonian liquid food in a tubular heat exchanger. Chem. Eng. Commun..

[2] Zamzari F, Mehrez Z, Cafsi AE (2017). Numerical investigation of entropy generation and heat transfer of pulsating flow in a horizontal channel with an open cavity. J. Hydrodyn..

[3] Ji J-D, Ge P-q, Bi W-b (2018). Numerical analysis of shell-side flow-induced vibration of elastic tube bundle in heat exchanger. J. Hydrodyn..

[4] Zhu Y, Zeng Q, Wan L, Yang Y, Li Z (2023). Vibration response difference of caving mechanism under coal rock impact based on mechanical–hydraulic coupling. Sci. Rep..

[5] Bhutta MMA, Hayat N, Bashir MH (2012). CFD applications in various heat exchangers design: A review. Appl. Therm. Eng..

[6] Su Y, Li M, Liu M (2016). A study of the enhanced heat transfer of flow-induced vibration of a new type of heat transfer tube bundle—the planar bending elastic tube bundle. Nucl. Eng. Design.

[7] Ji J, Ge P, Bi W (2016). Numerical analysis on shell-side flow-induced vibration and heat transfer characteristics of elastic tube bundle in heat exchanger. Appl. Therm. Eng. Des. Processes Equip. Econ..

[8] Sadek O, Mohany A, Hassan M (2018). Numerical investigation of the cross flow fluidelastic forces of two-phase flow in tube bundle. J. Fluids Struct..

[9] Ding Z (2023). Influence of support gap on flow induced vibration of heat exchange tube. Ann. Nucl. Energy.

[10] Ricciardi G, Pettigrew MJ, Mureithi NW (2011). Fluidelastic instability in a normal triangular tube bundle subjected to air-water cross-flow. J. Pressure Vessel Technol.ogy.

[11] In-Cheol C, Heung JC, Seungtae L (2011). Flow-induced vibration of nuclear steam generator U-tubes in two-phase flow. Nucl. Eng. Design.

[12] Tang D, Bao S, Lv B (2019). Investigation of shedding patterns and its influences on lift performances of a cylinder bundle in cross flow. J. Mech. Sci. Technol..

[13] Tang D, Bao S, Luo L (2019). A CFD/CSD coupled method with high order and its applications in flow induced vibrations of tube arrays in cross flow. Ann. Nucl. Energy.

[14] Tang D, Bao S, Xu M (2019). On the number of tubes required to study oscillating vortices and frequency spectrums of tube arrays in cross flow. Ann. Nucl. Energy.

[15] Tan W, Hao Wu, Zhu G (2019). Investigation of the vibration behavior of fluidelastic instability in closely packed square tube arrays. Trans. Tianjin Univ..

[16] Ai S, Sun C, Liu Y (2022). Numerical simulation of flow-induced vibration of three-dimensional elastic heat exchanger tube bundle based on fluid-structure coupling. Shock Vib..

[17] Ding Z, Bai X, Zhai Y (2022). Numerical simulation research on the vibration of helical tube arrays under transverse flow. Energies.

[18] Ansys Inc (2011). Ansys Fluent Theory Guide.

[19] Bao M, Wang L, Li W, Gao T (2017). The vibration analysis of tube bundles induced by fluid elastic excitation in shell side of heat exchanger. IOP Conf. Ser. Mater. Sci. Eng..

[20] Hai Z, Puzhen G, Ruifeng T, Xiaochang L (2023). A three-dimensional refined numerical simulation of cross-flow induced vibration mechanism in the tube bundle. Nucl. Eng. Design.

[21] Darwish S, Hadji A, Pham HP (2022). Flow-induced vibrations of a rotated square tube array subjected to single-phase cross-flow. J. Pressure Vessel Technol..

[22] Balabani S, Yianneskis M (2016). An experimental study of the mean flow and turbulence structure of cross-flow over tube bundles. Proc. Inst. Mech. Eng. Part C J. Mech. Eng. Sci..

[23] Menter FR (1994). Two-equation eddy-viscosity turbulence models for engineering applications. AIAA J..

[24] Stergiannis N, van Beeck J, Runacres MC (2017). Full HAWT rotor CFD simulations using different RANS turbulence models compared with actuator disk and experimental measurements. Wind Energy Sci. Discuss..

[25] Upnere S (2018). Numerical study of flow-induced vibrations of multiple flexibly-mounted cylinders in triangular array. Latvian J. Phys. Tech. Sci..

[26] Su W, Tao K, Liu F (2023). Numerical analysis of vibration response of elastic tube bundle of heat exchanger based on fluid structure coupling analysis. Nonlinear Eng..

[27] Shahzer MA (2022). A comprehensive investigation of vortex-induced vibrations and flow-induced rotation of an elliptic cylinder. Phys. Fluids.

[28] GB, T 151–2014 (2014). Heat Exchanger.

[29] Meirovitch L (2001). Fundamentals of Vibrations.

[30] Zheng M, Han D, Gao S (2020). Numerical investigation of bluff body for vortex induced vibration energy harvesting. Ocean Eng..

[31] Sattari AS (2022). Study of wave propagation in discontinuous and heterogeneous media with the dynamic lattice method. Sci. Rep..

[32] Tewari A (2007). Atmospheric and Space Flight Dynamics.

[33] Zhang, J. P., & Pan, L. Three-dimensional modeling and aeroelastic coupling analysis for the wind turbine blade. In *2009 World Non-Grid-Connected Wind Power and Energy Conference*, 1–4 (IEEE, 2009).

[34] Hafeez A (2023). Analysis of flow-induced vibrations in a heat exchanger tube bundle subjected to variable tube flow velocity. Adv. Sci. Technol. Res. J..

[35] De Pedro B, Parrondo J, Meskell C (2016). CFD modelling of the cross-flow through normal triangular tube arrays with one tube undergoing forced vibrations or fluidelastic instability. J. Fluids Struct..

[36] Tan W, Zhao C, Ren P (2022). Fluidelastic instability research of tube bundles by a two-way fluid-structure interaction simulation. Int. J. Pressure Vessels Piping.

[37] Joy A, Joshi V, Narendran K, Ghoshal R (2023). Piezoelectric energy extraction from a cylinder undergoing vortex-induced vibration using internal resonance. Sci. Rep..

[38] Lee YJ (2019). Vortex-induced vibration wind energy harvesting by piezoelectric MEMS device in formation. Sci. Rep..

[39] Qu Y (2023). Numerical study on vortex-induced vibrations of a flexible cylinder subjected to multi-directional flows. Phys. Fluids.

[40] Ozgoren M, Rockwell D (2007). Interaction of a deep-water wave with a vertical cylinder: Effect of self-excited vibrations on quantitative flow patterns. J. Fluid Mech..

